# An Introduction to Harm Reduction for Medical Students: Addressing Stigma Through an Interactive Preclerkship Lecture

**DOI:** 10.7759/cureus.44076

**Published:** 2023-08-24

**Authors:** Cynthia Janku, Lauren De Sa, Adegbemisola Daniyan

**Affiliations:** 1 Neurology, California University of Science and Medicine, Colton, USA; 2 Internal Medicine/Pediatrics, California University of Science and Medicine, Colton, USA; 3 Education, California University of Science and Medicine, Colton, USA

**Keywords:** persons who use injection drugs, community health services, medical student survey, harm reduction, medical student training, medical education curriculum

## Abstract

Introduction: Harm reduction is a non-traditional approach to addressing substance use disorders and a tool to prevent the spread of transmissible blood-borne infections. We taught an interactive lecture on harm reduction for medical students at the California University of Science and Medicine. This lecture was unique in that it is the only one that was directly developed in collaboration with a harm reduction nonprofit organization for the purpose of educating future physicians.

Methods: The class was encouraged to think critically about the topic of harm reduction, their biases toward persons who use injection drugs (PWID), and the role of physicians in improving health outcomes for this population. We sent pre- and post-surveys to the students to measure changes in their attitudes toward PWID and harm reduction.

Results: Overall, we successfully changed medical students' perspectives of PWID. However, their perspectives on the topic of harm reduction did not change significantly from the already positive opinions students had on the topic before the session. After the session, students were less likely to enjoy giving extra time to these patients and were more likely to find that these patients were difficult to work with.

Discussion: Harm reduction interventions can potentially prevent health complications associated with drug use, such as bacterial endocarditis, abscess formation, and transmitting diseases such as hepatitis and HIV, alleviating some of the burden placed on healthcare systems by PWID. This interactive session can serve as a model for other institutions that desire to educate their medical students on the topic of harm reduction and to address the stigma that is faced by PWID.

## Introduction

The AIDS epidemic of the 1980s significantly influenced the evolution of harm reduction in the United States [1]. It was during this time that harm reduction began to be viewed not only as an approach to addressing substance use disorders but as a tool to prevent the spread of transmissible blood-borne infections. A large proportion of people who were infected with HIV/AIDS were people who used injection drugs (PWID). Consequently, healthcare professionals and harm reduction activists recognized the need for collaboration with each other [1]. Syringe exchange programs and safer sex kits, two harm-reduction-focused interventions, soon proved to be an important part of curbing the spread of disease among people who engaged in high-risk practices. Despite this history, the harm reduction model has not yet been widely integrated into medical school curricula. The harm reduction model is predicated on the assumption that persons who engage in risky behaviors will continue to engage in those same behaviors and that interventions should focus on reducing the harm associated with those behaviors. Applying sunscreen to protect against UV radiation, wearing a seatbelt for protection in a car, and using earplugs at concerts to prevent hearing loss are all examples of harm reduction that people practice regularly [2]. Harm reduction in the context of drug use can include needle exchange programs, drug testing kits that detect fentanyl, and Narcan distribution to prevent opiate overdose, among other services.

An important aspect of harm reduction education is to dismantle the stigma that exists for marginalized groups. Currently, very few medical schools have incorporated formal pre-clinical training for medical students, pertaining to the stigma that exists among healthcare professionals toward patients with a substance use disorder. Link and Phelan defined stigma as the processes of stereotyping, labeling, separation, status loss, and discrimination co-occurring to have deleterious effects on financial earnings, housing, health, and life itself [3]. The lack of substance use training in medical education has been linked to negative attitudes toward people with opioid use disorder (OUD) [4]. These attitudes often persist throughout residency and post-residency, having important implications for OUD treatment practices, policies designed to expand access to OUD medications, and overall clinical care for people with a substance use disorder. A national survey of United States primary care physicians (PCPs) indicated that PCPs have high rates of stigmatizing attitudes in their profession [5]. Less than 30% of PCPs indicated that they would be willing to have a person who was being treated medically for an OUD as a neighbor or relative by marriage. Increased stigma was associated with lower support for policies that promoted increased access to OUD medication and an 11% lower likelihood that PCPs prescribed OUD medication [5]. This study suggests that the pervasive stigma existing among physicians directly impacts health outcomes for people with substance use disorders.

We developed an educational intervention designed to provide an introduction to the topic of harm reduction for second-year medical students at the California University of Science and Medicine. The session took place during the two-hour period that is set aside for College Colloquium, a weekly course run by the university that focuses on topics that lie at the intersection of medicine, ethics, and social issues. This interactive lecture for medical students was unique in that it was the only one of its kind to be directly developed in collaboration with a harm reduction nonprofit organization. We sought the expertise of a local organization called Inland Empire Harm Reduction (IEHR) because we wanted the lecture to be informed by the perspectives of PWID and the staff who work closely with them at IEHR. This organization serves clients in San Bernardino and Riverside counties in the United States. The objectives of the lecture were to provide an overview of the concept of harm reduction and to encourage students to reflect on the impact of their own stigmatizing beliefs on the morbidity and mortality of patients.

Different parts of the data in this article were previously presented at the California University of Science and Medicine Research Symposium on April 28, 2023. 

## Materials and methods

The Institutional Review Board of California University of Science and Medicine granted approval (HS-2021-12) to create and host a Colloquium session and collect pre- and post-survey results.

Prior to the lecture, students were asked to participate in an anonymous survey. Loma Linda University published a study in 2002, where they found that medical students had lower regard for patients who use drugs, especially injection drugs, compared to patients with diseases such as pneumonia, heartburn, and major depression [6]. This study validated the scaling tool of the Medical Condition Regard Scale (MCRS) as a way of measuring the attitudes and biases of medical students in training. The survey used in the current study consisted of questions taken from the MCRS and also asked students about prior experiences during which they may have worked with PWID [7].

During the Colloquium session, the class was encouraged to think critically about the topic of harm reduction, their biases toward PWID, and the role of physicians in improving health outcomes for this population. We had a presentation to discuss the concept of harm reduction and how that could be incorporated into patient care. We discussed our own relation to PWID, the pipelines to drug use, and the statistics of Riverside and San Bernardino County drug use to emphasize the importance of understanding the community's prevalence of drug use. The Operations Specialist of IEHR spoke to the cohort about the community that the organization serves and the resources available to clients. She shared common concerns about physicians and accessing care that are prevalent among PWID. The Operations Specialist focused on how clients expressed dissatisfaction with their healthcare once they admitted to using injection drugs to their physicians. 

After the presentations, the class was broken into small groups to discuss their own viewpoints and biases towards PWID, harm reduction, and how future physicians can incorporate those ideas into their practice. Two videos were shown to the class, which demonstrated encounters between a physician and a patient who was seeking opioid medication. The first video provided an example of a positive interaction between the physician and the patient. The second video presented a negative encounter between the physician and the patient. Students were asked to critique both videos and to identify what the physician did well in the first video and what the physician could have improved upon in the second video to address the patient's addiction to opioids. Students were asked to challenge their beliefs on what offering harm-reduction resources can do for patients. After the session, we emailed out another anonymous survey to complete, which was similar to the pre-survey.

To measure the efficacy of our lecture on changing perceptions toward PWID and harm reduction, we collected the survey results and ran paired t-tests on the pre- and post-survey groups. 

## Results

The lecture was presented in person to the 125-person second-year class at California University of Science and Medicine. The pre- and post-surveys were voluntary and anonymous. We had 68 people complete the pre-survey and 22 people complete the post-survey. Overall, the lecture had a positive impact on students, changing their perspectives and knowledge regarding PWID (Table 1). 

**Table 1:** Mean Difference in Scores Among Participants on Perception of People Who Use Injection Drugs (pre-survey N=68, post-survey N=22) Rated on a 5-point scale (1 = Most Negative, 5= Most Positive)

Question	Pre-Survey Mean Score	Post-Survey Mean Score	Difference	P-value
How do you feel about people that use injection drugs?	3.12	3.45	0.34	0.05

Student perspectives on the topic of harm reduction did not change significantly, but harm reduction was positively viewed even prior to the class session (Table 2). 

**Table 2:** Mean Difference in Scores Among Participants on Perception of Harm Reduction Organizations (pre-survey N=68, post-survey N=22) *Rated on a 5-point scale (1 = Most Negative, 5= Most Positive) ^#^Rated on a 5-point scale (1: Safe sex supplies (free lubrication/condoms), 2: Providing local resources for homeless shelters/treatment centers, 3: Safe used-needle disposal, 4: Providing clean needles, 5: Providing alternative use kits (i.e. snorting kits, etc)) ^Rated on a 5-point scale (1 = Does Not Support Harm Reduction, 5= Support All Levels of Harm Reduction)

Question	Pre-Survey Mean Score	Post-Survey Mean Score	Difference	P-value
How do you feel about harm reduction organizations?*	4.55	4.68	0.13	0.20
I support harm reduction up to and including:^#^	4.42	4.73	0.31	0.17
Do you consider yourself a harm reductionist?^	4.31	4.36	0.05	0.11

The students' perspectives on this population did not change significantly other than that students are less likely to enjoy giving extra time to these patients and finding that these patients are difficult to work with. After the presentation, students were more inclined to agree that insurance plans should cover PWID to the same degree as patients who live with other medical conditions (Table 3). 

**Table 3:** Mean Difference in Scores Among Participants on Perception of People Who Use Injection Drugs as Patients (pre-survey N=68, post-survey N=22) Rated on a 5-point scale (1 = Most Negative, 5= Most Positive)

Question	Pre-Survey Mean Score	Post-Survey Mean Score	Difference	P-value
I prefer not to work with patients like this.	1.70	2.00	0.30	0.16
Patients like this irritate me.	1.68	1.95	0.28	0.16
I enjoy giving extra time to these patients.	3.43	3.18	-0.25	0.03
Patients like this are particularly difficult for me to work with.	2.55	2.64	0.08	0.02
Working with patients like this is satisfying.	3.64	3.73	0.09	0.07
I feel especially compassionate towards patients like this.	3.96	3.82	-0.15	0.11
I wouldn’t mind getting up on night call for patients like this.	3.71	3.55	-0.17	0.08
I can find something to help patients like this feel better.	3.11	3.27	0.17	0.23
There is little I can do for patients like this.	2.20	1.86	-0.33	0.06
Insurance plans should cover patients like this to the same degree that they cover patients with other conditions.	4.09	4.23	0.14	0.05
Treating patients like this is a waste of medical dollars.	1.30	1.50	0.20	0.26

## Discussion

Harm reduction interventions such as clean needles, injection supplies, Narcan, and fentanyl test strips can potentially reduce the burden on healthcare systems by decreasing the spread of transmissible disease and complications from using contaminated supplies. Furthermore, many harm reductionists view healthcare in a positive light and have the potential to bridge the gap between healthcare providers and PWID. For example, these nonprofits teach people how to practice wound care and educate them on the importance of seeking medical treatment when necessary. Outside the realm of drug use, IEHR has designed programs to address the anti-vaccination sentiment among this population during the coronavirus disease 2019 (COVID-19) pandemic. Harm reduction can be an invaluable tool to the medical community by focusing on the prevention of overdose, decreasing the likelihood of abscess formation, and spreading basic health knowledge within the sensitive population of PWID.

Another centerpoint of our class discussion was that an approach focused on abstinence among PWID has been unsuccessful thus far. The United States continues to see a rise in overdose deaths nationally [8]. PWID are less likely to seek abstinence when they are negatively perceived by healthcare providers. The interactive lecture we provided to a group of future physicians encouraged students to consider alternatives to abstinence, given that not all PWID are immediately seeking abstinence. Medical students were given the opportunity to reflect on the words they recited when they took the Hippocratic oath: "I will maintain the utmost respect for human life.” Regardless of their willingness to abstain from drug use, PWID should have access to high-quality healthcare and safe interactions with their physicians. 

Current literature reflects that pre-exposure prophylaxis in PWID can prevent HIV transmission, but many medical providers are not educated on available local resources and barriers that patients face in receiving care [9]. In a study on patients with infective endocarditis conducted in a tertiary care center in Maine, 39.2% had infective endocarditis associated with unsafe injection use practices [10]. Of course, many challenges have been faced since the COVID-19 pandemic, increasing the difficulty to access resources and decreasing the ability to provide outreach services [11,12]. Dietze and Peacock discussed how the COVID-19 pandemic increased harm to PWID due to decreased social avenues for safety [13]. In such a scenario, people are more likely to use drugs alone, leading to increased overdose-related deaths. Promoting harm reduction as physicians can allow for decreased stigma and increased awareness in this population, even in times of decreased social networking and resource availability. 

At least 69 out of 133 accredited United States medical schools in 2011 had incorporated a medical humanities course into their curricula [14]. That number has most certainly increased over the years. The content presented during the interactive session at the California University of Science and Medicine can be adapted to fit into these medical humanities courses. The data gathered from our pre- and post-surveys suggests that just one session on the topic of harm reduction was able to change student perspectives on PWID. By implementing such a session into their curricula, medical schools have the ability to develop a new generation of physicians who will be sensitive to the healthcare needs of PWID and supportive of the role that harm reductionists play in decreasing the negative effects of substance use.

Limitations

One limitation of our study is the discrepancy between the number of students who completed the pre-survey and the number of students who completed the post-survey. Challenges to our interactive lecture included losing students to follow-up and the delay in sending the post-survey. The post-survey was emailed 12 days after the class session due to Thanksgiving break and the concern that the post-survey would not be seen during the break. The decision to email the survey after the break may have resulted in lower post-survey responses. While we have not identified any obvious differences between respondents and non-respondents, it is possible that differences between the groups exist and have contributed to nonresponse bias. Another limitation of this study is that the session was largely focused on local details relevant to the Inland Empire, possibly limiting the generalizability of our study to other contexts and settings.

## Conclusions

Harm reduction interventions can potentially prevent health complications associated with drug use, alleviating some of the burden placed on healthcare systems by PWID. Harm reduction can be an invaluable tool to the medical community by focusing on the prevention of overdose, decreasing the likelihood of abscess formation, and spreading basic health knowledge within the sensitive population of PWID. This interactive class session improved students' perceptions of PWID and also informed students of local resources available to this vulnerable population. This interactive session can serve as a model for other institutions that desire to educate their medical students on the topic of harm reduction and to address the stigma that is faced by PWID.
